# Physical Activity in 15–17-Year-Old Adolescents as Compensation for Sedentary Behavior in School

**DOI:** 10.3390/ijerph17093281

**Published:** 2020-05-08

**Authors:** Lukáš Jakubec, Karel Frömel, František Chmelík, Dorota Groffik

**Affiliations:** 1 Faculty of Physical Culture, Palacký University Olomouc, 779 00 Olomouc, Czech Republic; karel.fromel@upol.cz (K.F.); frantisek.chmelik@upol.cz (F.C.); 2 Institute of Sport Science, The Jerzy Kukuczka Academy of Physical Education, 40-065 Katowice, Poland; d.groffik@awf.katowice.pl

**Keywords:** ActiTrainer, adolescent, intensity, physical inactivity

## Abstract

The traditional concept of education and school settings significantly contribute to the sedentary behavior of adolescents at secondary schools. The aim of this study is to identify the volume and intensity of physical activity (PA) that adolescent boys and girls engage in during recesses, after school, and during the day to compensate for sedentary behavior in lessons. The study was conducted at 29 Czech and 9 Polish schools. The study involved 868 girls and 409 boys aged 15–17 years. An ActiTrainer^TM^ accelerometer was used to monitor PA and heart rate. Participants were divided into four quartile groups. Most sedentary boys and girls had less PA and showed a worse ratio of physical inactivity (PI)/PA than non-sedentary participants during recesses. In the after-school period, there were no significant differences. On school days, most sedentary boys and girls showed lower PA, a worse ratio of PI/PA, fewer steps·hour^−1^, and lower energy expenditure than their non-sedentary counterparts. Vigorous PA of ≥8 METs was reached by 48% of most sedentary boys (75% non-sedentary) and 47% of most sedentary girls (54% non-sedentary). Most sedentary adolescents do not compensate for their sedentary behavior in lessons with higher PA intensity or volume during recesses, after-school, or in overall daily PA.

## 1. Introduction

School time in relation to the increasing academic requirements [1,2] and the effort to achieve the highest possible academic achievement [3] significantly contributes to sedentary behavior (SB) in adolescents [4]. We understand the SB in accordance with the terminological consensus according to Tremblay et al. [5] where it was defined as any waking behavior characterized by an energy expenditure ≤1.5 metabolic equivalents, while in sitting, reclining, or lying posture. The negative effects of SB on physical health are well documented [6,7,8], as are the negative trends in the prevalence of SB [1,9,10,11]. Schools cannot waive their responsibility for controlling adolescents’ SB in the school environment [12]. Therefore, the WHO European physical activity strategy for 2016–2025 requires governments and public policies to support active commuting, such as walking and cycling, to school and from school, and to ensure that school curricula for adolescents include a strong physical education lesson (PEL) component [1]. Similarly, in the USA there are numerous national initiatives to increase adolescents’ active transport to and from school, and to increase the amount of time youths spend engaging in moderate-to-vigorous physical activity (MVPA) during physical education lessons [13]. During school time, the greatest compensation effect of adolescents’ SB is expected from high-quality physical education lessons (PELs).

PELs should incorporate at least 50% of moderate-to-vigorous physical activity (MVPA) lesson time [14]; however, this is achieved by fewer than half of all adolescents [15]. Meta-analytic studies suggest that high school students spend on average only 35.9% of MVPA time in PELs [16]. It is also recommended that children’s recesses include at least 50% MVPA [17] and in high schools together with previous lessons on average 500 steps/hour [15,18]. There is a sufficient body of data on physical activity (PA) and SB during recesses in children [19]. Unfortunately, less information is available about the quantity and intensity of PA and SB during recesses in high school students [20,21,22]. However, also in high schools, PELs and recesses have a crucial effect on PA and SB compensation [15,16,22,23,24]. Schooldays with PELs increase daily MVPA as opposed to days without PELs [25,26,27]. A higher number of weekly PELs in Poland compared with a lower number in the Czech Republic is significantly associated only with higher weekly vigorous PA [28].

In addressing the issue of SB in secondary schools it must be considered that MVPA decreases and SB increases with age [1,29,30]. It means that it is essential to respect the differences in PA and SB of boys and girls according to the years at secondary schools. In particular, the transition to a higher educational level is a significant period during which PA decreases [31]. At the same time, it is known that most high school adolescents fail to meet the current 24-Hour Movement Guidelines for Children and Youth [13,32,33,34,35]. It is also known that the achievement of these guidelines and the overall level of daily and weekly PA in adolescents are also significantly dependent on the level of school PA [36,37]. In Central Europe, school PA assessed by step counts represents on average 23%–39% of the overall daily PA [28] and 29% according to MVPA [15]. 

Unfortunately, existing studies on SB and PA do not provide any evidence of the compensation effect of PA on SB and cognitive load of students in school. It is assumed that any health-oriented PA in school and after school has a compensation effect. An increase in MVPA in school increases overall daily MVPA on schooldays, but there is no evidence whether or not MVPA decreases in the following days [37]. Nor is it clear whether or not a greater compensation effect on SB and students’ cognitive load in school is brought by a greater amount of PA or higher PA intensity, immediate shorter PA or delayed physically demanding PA, or a combination of PA with other relaxation techniques. This study should be an impetus for solving this current problem of supporting the adolescents’ health during their time spent at school.

It is assumed that the adoption of the habit of compensating for sitting by immediate PA in school irrespective of the volume or intensity of PA provides the basis for a future occupational and lifelong healthy lifestyle. This study focuses on adolescents who belong to the group with high-risk sedentary behavior, which affects their physical health.

The aim of this study is to identify the volume and intensity of physical activity among adolescent boys and girls during recesses, after school, and during the day to compensate for sedentary behavior in lessons. Further to find differences in volume and intensity of PA between non-sedentary and most sedentary boys and girls. 

## 2. Materials and Methods 

### 2.1. School Setting and Participants

The research was conducted at 29 Czech and 9 Polish secondary schools during the fall season. The participating schools were selected according to the place of residence of the undergraduate students who were at the schools as student teachers at the time of the research or later. Proper habitual week without any disruption of regular school schedule was selected with the school management if the school agreed to participate in the study. The size of involved schools was about 50 to 80 students in every year of study. All the selected schools had a similar organization education process: classes usually start at 8 a.m. and end between 2 and 3 p.m. Each class lasts 45 min. The recess after the second lesson of the day lasts 20 min, the duration of other recesses varies from 5 to 10 min. In addition, if there was a recess for lunch, the recess lasted at least 30 min. During school recesses and lunch recesses, the participants could move freely on campus and had the opportunity to use school facilities for various unstructured physical activities. Students used those facilities very rarely. None of the schools had implemented a special PA program intended for recesses, lunch recesses, or for carrying out lessons in subjects other than PELs. None of the schools used special equipped rooms except PELs. One of the typical movement behaviors during the recesses was to move from one classroom to another, but all classrooms were in the same building. 

In total, 2702 students aged 15 to 17 years old participated in the study. Due to incomplete record sheets or insufficient wear time, 959 participants were excluded. Inclusion criteria were set as follows: for at least 15 min before classes, at least 180 min at school (excluding PEL), at least 120 min after school, and at least 600 min per day. An additional 466 participants were excluded from the study because of having PEL in their daily school schedule. School days with PEL were excluded because participation in PELs could negatively affect the character of movement behavior during recess before and after PELs. Simultaneously, results that could be distorted when students who do not participate in PELs from various reasons are included in the data sample. No swimming was reported in the participants’ daily PA. Finally, 868 girls and 409 boys (response rate 47.26%) met the criteria to participate in the study (Table 1). Only 6.7% of boys and 9.3% of girls were categorized as overweight and obese [≥85th percentiles of body mass index (BMI)] based on WHO BMI percentile charts. All participants and their parents agreed to be involved in the study and provided written informed consent (response rate = 95%). Disagreement with the study occurred exceptionally, typically in one or two students per class. Boys and girls were divided into four quartile groups according to the ratio between the time of physical inactivity (PI) and the time of PA during classes. This unusual approach for dividing participants according to the PI/PA ratio is because the ActiTrainer^TM^ (Pensacola, FL, USA) accelerometer can precisely capture PI (defined as <100 counts per minute) and on top of that the total amount of PA captured by the accelerometer is more reliable than dividing the groups according to the particular levels of PA. For the purpose of this study we defined PI as a physical activity that is lower than 100 counts per minute. This definition differs from the definition of PI presented in Tremblay et al. [5] because it captures different behavior. The following groups were created for further analyses: non-sedentary (NS), little sedentary (LS), much sedentary (MS), and most sedentary (S). The study focuses on differences between the S and the NS student group.

The study was approved by the Institutional Research Ethics Committee of the Palacký University Olomouc (decision no. 24/2012).

### 2.2. Instruments

The ActiTrainer^TM^ accelerometer was used to objectively assess PA. This device is a lightweight and compact triaxial accelerometer (8.6 × 3.3 × 1.5 cm, 51 g) designed to measure the amount and frequency of human activity: activity counts and vector magnitude, energy expenditure, steps taken, activity intensity levels, and subject position. The device also collects heart rate (HR) data when the Polar^TM^ chest strap (type S610 was used in this study) is worn across the sternum. Our department verified the device’s validity of step count in field conditions. The correlation values expressing the relationships between actual and device-measured steps ranged from 0.96 to 0.97 [38]. Accelerometers have acceptable reliability and validity in pediatric populations [39,40,41]. ActiLife software version 6.13.2 (ActiGraph^TM^, Pensacola, FL, USA) was used for device initialization and data downloading. The epoch duration was set at 15 s.

### 2.3. Procedure

Participants used the accelerometer to monitor their PA and HR for three consecutive school days of the same week from morning (after morning hygiene) throughout the day to the evening (hygiene before bedtime). Participants wore the device on their right hip and the chest strap across the sternum and took it on and off when changing their clothes. They were not allowed to wear the device during water activities (e.g., swimming, showering, bathing). Individual resting HR was measured in accordance with the exact instructions in the morning after waking up, repeatedly three times in 15-s intervals. The measured values for each measurement were converted to the number of beats per minute and recorded on the record sheet. Individually measured HR resting values were used to calculate the mean value that was, in the case of higher values, corrected according to the lowest daily HR value as measured by the accelerometer. During the day, participants recorded the following times: putting the devices on, arrival at school, school hours including school breaks, departure from school, participation in organized PA, and taking the devices off before bed. All instructions were provided by the research team during the initial session. Researchers obtained participant’s date of birth, body weight using Tanita^TM^ weight scale (UM-075 type; Tanita Corporation, Tokyo, Japan) and height using Leicester height measure during this session. PA monitoring starts in the morning of the following day. After three or five weeks from the end of the monitoring, all participants received the following feedback: time data on PA and PI, energy expenditure, HR, and step counts. The feedback also included information on load in METs and HR zones, together with clear energy expenditure curves, and HR curves. Individual and group results were analyzed as a part of biology lessons at schools.

Data processing from the ActiTrainer^TM^ accelerometer followed the same procedure as previous studies that used the same methods [15,18,42]. A specially created software IntPA13 (https://upol.cz/fileadmin/userdata/FTK/Fakulta/Verejnost/Navod_IntPA13.pdf; SoftWareCentrum, Olomouc, Czech Republic) was used to process the 15-s epoch accelerometer data. The software enables us to evaluate data in different parts of the day: before school, at school, after school, during recesses, in PELs, and over the whole day. The ranges of load were determined according to performed steps, HR in 30%–100% HRmax in 10% intervals and in METs as a metabolic equivalent in 1 MET interval. The intensity zones were classified individually as follows: low (LPA; 50%–59.9% HRmax; <3 METs), moderate (MVPA; 60%–84.9% HRmax; 3–5.9 METs), or vigorous PA (VPA; ≥85% HRmax; ≥6 METs). The sedentary (physical inactivity) cut points were set to <25 counts per 15 s. Physical inactivity is understood as unrecorded change of the “center of gravity” of the body, with up to 100 counts per minute, simply as a “stable body position” in sitting, lying, or a different body position [15]. All of the data were converted into relative values reflecting the wear-time of the accelerometer in each part of the day.

Further, processed accelerometry data were compared with guidelines for the physically active lifestyle of adolescents based on the present published literature [14,15,43,44,45] for step count and PA in different parts of the school day as follows: 1000 steps (set according to the median of performed steps) and at least 50% of PA during recesses per school day; 6000 steps and least 30 min of MVPA after school per school day; and 11,000 steps and 60 min of MVPA in total per school day.

In compliance with the main aim of the study, the before school segment is not presented in this paper, but the differences between the groups were not significant in this segment.

### 2.4. Data Analysis

In Statistica version 13 (StatSoft, Prague, Czech Republic) and SPSS version 25 (IBM SPSS, Inc., Chicago, IL, USA) programs, we used descriptive statistics for basic characteristics of the sample. Crosstabulations were used to undertake a group assessment of whether the recommendations for PA were met. Kruskal–Wallis ANOVA was used to ascertain the differences among groups with different proportions of PI and PA (quartile groups). Further to compare chosen pairs, multiple comparisons of means rank was used for all groups as a post-hoc test. Binary logistic regression with the enter method (all independent variables are entered into the equation at the same time) was used to find out the odds ratio in meeting PA recommendations between the non-sedentary and sedentary adolescents. Further, the *ŋ*^2^ and *r* effect size coefficients were calculated. The effect size coefficients were interpreted as follows: small effect size *ŋ*^2^ = 0.01–0.059, r = 0.1–0.29; medium effect size *ŋ*^2^ = 0.06–0.139, r = 0.30–0.49; large effect size *ŋ*^2^ ≥ 0.14, r > 0.50. Significance was set at *p* < 0.05.

## 3. Results

### 3.1. Compensation for Sedentary Behavior during Recesses, after School, and Whole Day with PA

#### 3.1.1. Recesses

Non-sedentary boys and girls spent significantly more time in LPA according to METs (*p* < 0.001, resp. *p* = 0.021) during recesses than their sedentary pears (Table 2). Higher level of PA in non-sedentary boys and girls was also confirmed by the significantly different ratio of PI/PA (*p* < 0.001, resp. *p* < 0.001). Further, non-sedentary boys had a significantly higher number of steps (*p* = 0.020) and caloric expenditure (*p* = 0.001) than sedentary boys. The differences in physical load according to HR and time spend in MVPA according to METs were not significant between non-sedentary and sedentary participants. During recesses, only 7% of non-sedentary boys (7% of sedentary) and 5% of non-sedentary girls (2% of sedentary) reached the load of ≥85% HRmax. The physical load of ≥8 METs was achieved by 18% of non-sedentary boys (6% of sedentary) and 8% of non-sedentary girls (5% of sedentary). Furthermore, we found that there are significant differences between non-sedentary boys and non-sedentary girls according to the time spent in LPA (*p* = 0.012) and in the ratio of PI/PA (*p* = 0.002). Finally, non-sedentary boys spent 9.0% of the recess time in MVPA (7.1% of sedentary), and non-sedentary girls spent 7.5% of their time in MVPA (5.7% of non-sedentary).

#### 3.1.2. After School

In the after-school segment, non-sedentary boys spent significantly more time in LPA according to METs (*p* = 0.012) and had a significantly better ratio of PI/PA (*p* = 0.017) in comparison to sedentary boys. We found no significant differences between non-sedentary and sedentary boys and girls in other characteristics of after-school PA. An HRmax load of ≥85% was achieved by 38% of non-sedentary boys (27% of sedentary) and by 32% of non-sedentary girls (32% of sedentary). The load of ≥8 METs was detected in 63% non-sedentary boys (39% of sedentary) and in 37% non-sedentary girls (41% of sedentary).

#### 3.1.3. Daily PA

Over the whole day, non-sedentary boys and girls showed more time spent in LPA according to METs (*p* < 0.001, resp. *p* < 0.001), a better ratio of PI/PA (*p* < 0.001, resp. *p* < 0.001), performed a higher number of steps (*p* < 0.001, resp. *p* < 0.001), and had higher energy expenditure (*p* < 0.001, resp. *p* < 0.001) than their sedentary peers. Results also showed that sedentary boys spent significantly more minutes in MVPA than sedentary girls (*p* = 0.014). In this study, 50% of non-sedentary boys (37% of sedentary) and 44% of non-sedentary girls (37% of sedentary) achieved a load of ≥85% HRmax during the day. The load of ≥8 METs was reached by 75% of non-sedentary boys (48% of sedentary) and 54% of non-sedentary girls (47% of sedentary). In total, the characteristics of daily PA showed that non-sedentary young people compensate for PI in classes more than the sedentary participants. Also, the daily results of the HR record correspond with the PA record. However, the differences between non-sedentary and sedentary participants were not significant.

### 3.2. Meeting the Recommendations in the Segments of School Day

#### 3.2.1. Steps Count and PA Intensity

**Figure 1:**
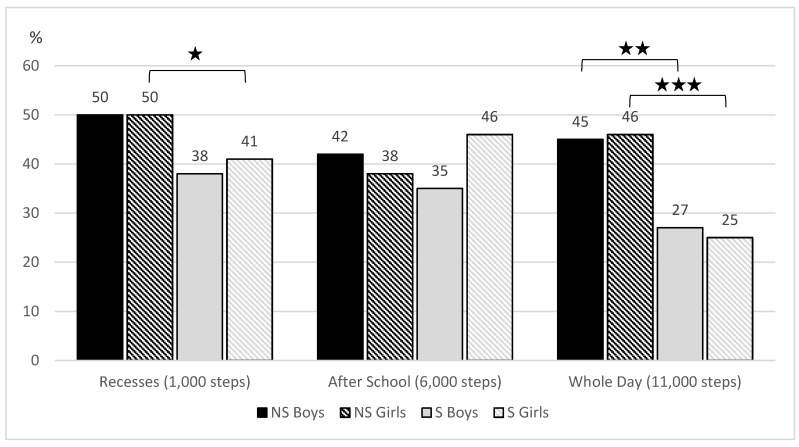
Meeting the guidelines for step count in different day segments (1000 steps/day during school recesses, 6000 steps/day after school, 11,000 steps/day per school day) among non-sedentary and sedentary boys and girls in classes. ★ *p* < 0.05; ★★ *p* <.01; ★★★ *p* < 0.001.

**Figure 2:**
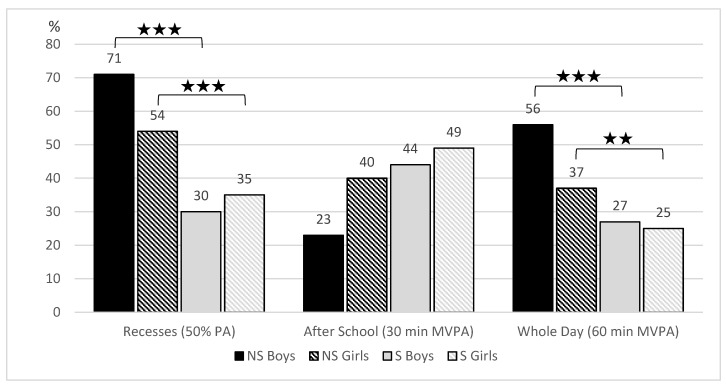
Meeting the guidelines for physical activity (PA) in different day segments (50% of PA during school recesses, 30 min of moderate-to-vigorous physical activity (MVPA) after school, 60 min of MVPA per school day) among non-sedentary and sedentary boys and girls in classes. ★ *p* < 0.05; ★★ *p* < 0.01; ★★★ *p* < 0.001.

Significant differences in meeting the steps recommendations (Figure 1) were found between non-sedentary and sedentary boys and girls in whole day (*χ*^2^ = 7.57, *p* = 0.006, *r* = 0.185 *, resp. *χ*^2^ = 19.49, *p* = < 0.001, *r* = 0.209 *) and during recesses in girls (*χ*^2^ = 4.10, *p* = 0.043, *r* = 0.096). Meeting the recommendations for PA according to the time and intensity of PA (Figure 2) corresponds with the step results. Statistically significant differences were found between non-sedentary and sedentary boys and girls in recesses (*χ*^2^ = 35.22, *p* < 0.001, *r* = 0.413 **, resp. *χ*^2^ = 14.91, *p* < 0.001, *r* = 0.180 *), and over the whole day (*χ*^2^ = 17.80, *p* < 0.001, *r* = 0.288 *; resp. *χ*^2^ = 7.84, *p* = 0.005, *r* = 0.127 *). No significant differences were found among the groups in the after-school segment.

#### 3.2.2. Predictors to Meet the PA Recommendations. 

Non-sedentary boys and girls have higher odds ratio to meet the daily step recommendation (Table 3) than their sedentary peers (*OR* = 2.38, *CI* = 1.30–4.34, *p* < 0.001, resp. *OR* = 2.47, *CI* = 1.64–3.71, *p* < 0.001) with adjusting for age, BMI, and country (Table 3). Non-sedentary boys and girls are also more likely to undertake at least 60 min of MVPA per day than their sedentary counterparts (*OR* = 3.62, *CI* = 1.98–6.63, *p* < 0.001, resp. *OR* = 1.82, *CI* = 1.20–2.76, *p* = 0.005).

## 4. Discussion

In the agreement with the aim of the study, we identified the volume and intensity of PA between boys and girls with different levels of SB in recesses, after school and in the whole day. The most important finding of the research is that boys and girls with the highest proportion of sedentary behavior do not compensate for sitting in lessons by means of PA volume or shorter higher-intensity PA in immediately subsequent recesses more than those who are less sedentary. The same applies to delayed PA compensation after school and to overall PA and PI indicators for the whole day. According to previous findings in similar conditions, students do not compensate for sedentary behavior either in school or at the weekend [46]. This emphasizes the importance of the development of appropriate conditions for a healthy school lifestyle in all students [47], especially in terms of supporting PA and decreasing sedentary time during school, both in children and adolescents [48,49,50]. A meta-analysis study by Egan et al. [50] revealed that school youth spend on average 63% of school time sitting. In the present sample, it was observed that in total boys spent 70% (girls 74%) of school time in PI. It is clear that even schools must start to respect the fact that decreasing any type of sedentary time is associated with a lower health risk in youth [51]. The fact that no significant differences were observed between sedentary and non-sedentary adolescents in PA after school corresponds with some interventions to increase school PA, which had no effect on free time after school [52].

Schools have the greatest chance to answer the simple but apt question according to Bouchard, Blair, and Katzmarzyk [53]: “Less sitting, more PA or higher fitness?” or to follow the simplified recommendation that applies to all population groups: “Move more and sit less” [54]. School strategies should include all of the three components of a physically active school lifestyle, but the greatest chance for schools is probably to reduce sitting time. Reducing sitting time of all students should also result in overall higher school PA in students without exception. In terms of educational organization, the differences in students’ sedentary behavior may be decreased, for example in schools where students need to move to special rooms. A focus on reducing sitting time in all students may also contribute to the elimination of gender differences in PA. Research studies confirm less sitting time on schooldays and higher PA in boys as opposed to girls [55,56]. The fact that PA indicators by HR do not show significant differences between boys and girls needs to be interpreted with respect to their different physiological responses. Even a difference of 2.3 beats/min in resting HR in the sample of the present study highlights the need to respect these gender differences.

The achievement of PA recommendations expressed by steps in recesses, after school, and over the whole day confirms insufficient compensation for SB in the most sedentary boys and girls. The determination of these recommendations was based on internationally acknowledged PA guidelines [14,45] taking into account the specificities of Central European conditions [15,18,44], and the most commonly applied recommendation on daily step counts according to Tudor-Locke et al. [43]. The achievement of PA recommendations in recesses (at least 50% PA), after school (at least 30 min of MVPA), and whole-day PA (at least 60 min of MVPA) in sedentary and non-sedentary boys and girls highlights, together with the step count recommendation, insufficient compensation for SB by means of MVPA. It appears that the evaluation of the compensation effects of PA by means of the simplest indicator such as step counts in daily segments may be simple, sufficiently informative, and well applicable in the school environment [57,58]. It is also important that girls are more motivated than boys to perform additional PA based on information regarding the number of steps [59]. The results of this study also supported the thought that the preference of the simple step count recommendation should not diminish the importance of others no less important PA where lower number of steps are performed.

The fact that, according to SB, non-sedentary boys and girls in school lessons have a greater chance of achieving the recommendation on daily PA present a challenge to reduce sitting for all students in the process of education, especially the most sedentary ones. Moreover, it is important to support the transfer of students’ school PA to other segments of the school day and weekly PA structure, including weekend days [46]. This requires comprehensive approaches based on a socio-ecological model of change [60], but also on the model of adolescents’ physical, mental, social, and economic aspects of well-being [61], especially because in Central Europe a prevalence of depression symptoms was observed in more than 25% of adolescents [62]. Schools can use a wide range of physical, social, and environmental factors to increase adolescents’ PA and reduce SB [47], especially in girls [55]. This requires sweeping changes in curricular reductions, innovation of the traditional program of education, consistent introduction and streamlining of school physical activity programs, as well as supporting interventions that respect demographic, educational, socio-economic, and other specificities. Further, it is essential to ensure the participation of all students in PELs without exceptions in the Czech Republic and Poland [28].

Further research on SB and PA in high schools should be more integrated into the changes of the education system and should respect students’ opinions more [63] and actively involve them in intervention research in order to increase the number of “healthy schools”.

### Strengths and Limitations

The study is one of the few that monitors whole-day PA in adolescents in METs and by HR, except sleep time, involving a higher number of students in natural school conditions. Monitoring whole-day HR using chest belts in the school environment is increasingly difficult; therefore, it was not possible to realize the random sample selection. The high number of excluded participants was mainly caused by insufficient wear time in observed day segments and by losing the appropriate contact between the chest belt HR monitor and skin of the participants’ body for several minutes. In the future it will be necessary to improve the quality of HR monitoring by using wrist wearables. Another positive aspect of the study is accurate time monitoring of the school program with minute accuracy, which on the other hand requires individual time records kept by all participants. Given the complexity of the research, the respondents’ physical fitness was not examined and HRmax was determined according to universal formulas.

## 5. Conclusions

Boys and girls in high schools do not compensate for SB by means of school PA in recesses or after school. It has not been confirmed that boys or girls use shorter VPA to compensate for SB. School managers and teachers should as much as possible try to support the adoption of adolescents’ habit of compensating for sitting by any PA, even short PA. Any interruptions in sitting periods in lessons are of crucial importance to the negative implications of SB on adolescents’ health. 

## Figures and Tables

**Table 1:** Sample characteristics.

Characteristics	*n*	Age (years)		Weight (kg)		Height (cm)		BMI (kg·m^−2^)		HRrest (min)	
		*M*	*SD*	*M*	*SD*	*M*	*SD*	*M*	*SD*	*M*	*SD*
Boys	409	16.60	1.04	71.78	12.28	179.16	7.61	21.09	2.90	60.77	6.54
Girls	868	16.52	0.98	58.94	9.09	167.10	6.18	20.09	2.90	63.08	7.24

BMI—Body Mass Index; *M*—mean; *SD*—standard deviation; HRrest—resting heart rate.

**Table 2:** Physical activity of sedentary and non-sedentary boys and girls during recesses, after school, and over the whole day.

Characteristics of PA	Boys—Sedentary				Girls—Sedentary				*H*	*p*	*η* ^2^
	Non (NS) (*n* = 104)	Little (LS) (*n* = 100)	Much (MS) (*n* = 104)	Most (S) (*n* = 101)	Non (NS) (*n* = 217)	Little (LS) (*n* = 217)	Much (MS) (*n* = 217)	Most (S) (*n* = 217)			
	*Mdn* *(IQR)*	*Mdn* *(IQR)*	*Mdn* *(IQR)*	*Mdn* *(IQR)*	*Mdn* *(IQR)*	*Mdn* *(IQR)*	*Mdn* *(IQR)*	*Mdn* *(IQR)*			
Recesses											
Time 50%–59.9% HRmax (min·h^−1)^	9.57 (14.19)	6.90 (12.90)	6.72 (16.70)	7.00 (12.29)	10.67 (12.25)	9.33 (12.25)	8.10 (11.43)	8.36 (11.00)	9.48	0.220	0.007
Time ≥60% HRmax (min·h^−1)^	0.59 (4.73)	0.00 (4.23)	0.55 (2.45)	0.00 (1.50)	1.29 (5.00)	0.78 (3.71)	0.82 (3.23)	0.75 (2.50)	29.28	< 0.001	0.023 *
Time <3 METs (min·h^−1)^	19.14 (3.92)	14.90 (2.04)	11.14 (1.89)	7.33 (2.27)	16.87 (4.19)	12.17 (2.07)	9.11 (1.59)	5.60 (2.02)	79.19 ^a,b,c^	< 0.001	0.062 **
Time ≥3 METs (min·h^−1)^	2.00 (2.82)	0.90 (1.72)	0.50 (1.07)	0.25 (0.56)	2.10 (2.28)	0.67 (1.43)	0.38 (0.74)	0.11 (0.38)	36.59	< 0.001	0.029 *
Ratio of PI/PA	0.70 (0.69)	0.77 (0.55)	0.92 (0.63)	1.23 (0.77)	0.94 (0.92)	1.03 (0.80)	0.94 (0.58)	1.20 (0.87)	104.30 ^a,b,c^	< 0.001	0.082 **
Steps·h^−1^	1066 (661)	1038 (735)	960 (614)	839 (482)	889 (746)	867 (661)	1013 (620)	920 (613)	21.21 ^a^	0.004	0.017 *
Energy expenditure Kcal·kg^−1^·h^−1^	0.68 (.56)	0.61 (0.54)	0.58 (0.46)	0.51 (0.31)	0.55 (0.47)	0.51 (0.38)	0.56 (0.37)	0.47 (0.36)	43.63 ^a^	< 0.001	0.034 *
After school											
Time 50%–59.9% HRmax (min·h^−1)^	7.71 (10.06)	6.70 (8.46)	7.78 (10.15)	5.90 (8.81)	7.78 (9.31)	7.58 (8.68)	7.42 (7.42)	7.35 (7.70)	5.07	0.652	0.004
Time ≥60%HRmax (min·h^−1)^	2.12 (11.05)	2.73 (8.32)	2.76 (9.11)	1.17 (5.59)	2.24 (5.05)	3.25 (6.83)	2.81 (5.90)	2.36 (4.83)	13.47	0.061	0.011 *
Time <3 METs (min·h^−1)^	18.67 (9.80)	17.52 (8.29)	16.13 (8.95)	15.11 (7.47)	18.86 (9.70)	17.76 (8.65)	18.23 (7.55)	17.88 (6.97)	31.37 ^a^	< 0.001	0.025 *
Time ≥3 METs (min·h^−1)^	4.53 (6.04)	4.12 (4.97)	4.84 (3.70)	3.86 (3.88)	3.52 (4.40)	3.53 (4.03)	3.93 (3.59)	4.07 (3.62)	16.04	0.025	0.013 *
Ratio of PI/PA	1.47 (1.53)	1.68 (1.40)	1.73 (1.73)	1.97 (1.65)	1.62 (1.37)	1.68 (1.30)	1.59 (1.01)	1.70 (0.96)	15.84 ^a^	0.027	0.012 *
Steps·h^−1^	814 (761)	673 (588)	791 (523)	662 (489)	717 (574)	724 (613)	784 (549)	804 (499)	8.95	0.256	0.007
Energy expenditure (kcal·kg^−1^·h^−1)^	0.65 (0.64)	0.56 (0.51)	0.60 (0.42)	0.49 (0.43)	0.47 (0.43)	0.49 (0.42)	0.51 (0.41)	0.54 (0.36)	14.39	0.045	0.011 *
Whole day											
Time 50%–59.9%HRmax (min·h^−1)^	9.17 (8.02)	7.97 (7.08)	8.02 (7.61)	8.24 (6.21)	11.20 (7.33)	9.98 (6.85)	9.47 (6.56)	9.52 (6.44)	32.78	< 0.001	0.026 *
Time ≥60%HRmax (min·h^−1)^	3.94 (7.11)	3.44 (6.69)	3.70 (5.29)	2.42 (4.86)	4.34 (5.98)	4.26 (6.04)	4.48 (5.30)	3.35 (4.16)	26.03	< 0.001	0.020 *
Time <3 METs (min·h^−1)^	21.84 (5.78)	19.55 (6.07)	18.13 (4.62)	16.21 (4.21)	20.92 (6.30)	19.15 (4.90)	18.35 (4.76)	16.51 (4.15)	241.04 ^a,b,c^	< 0.001	0.189 ***
Time ≥3 METs (min·h^−1)^	5.48 (4.16)	4.75 (4.22)	4.33 (2.80)	4.83 (3.70)	4.56 (3.06)	4.32 (3.58)	4.28 (3.11)	3.82 (2.84)	34.45 ^d^	< 0.001	0.027 *
Ratio of PI/PA	1.53 (0.47)	1.77 (0.38)	1.78 (0.48)	1.89 (0.43)	1.65 (0.45)	1.74 (0.43)	1.79 (0.37)	1.89 (0.33)	127.41 ^a,b^	< 0.001	0.100 **
Steps·h^−1^	798 (422)	598 (327)	638 (316)	591 (286)	709 (338)	642 (325)	649 (287)	605 (251)	55.24 ^a,b^	< 0.001	0.043 *
Energy expenditure (kcal·kg^−1^·h^−1)^	0.57 (0.42)	0.47 (0.27)	0.44 (0.24)	0.40 (0.23)	0.46 (0.26)	0.41 (0.23)	0.42 (0.20)	0.39 (0.19)	75.81 ^a,b^	< 0.001	0.059 *

*Mdn*—median values; *IQR*—interquartile ranges; H—Kruskal-Wallis test; ŋ^2^—coefficient effect size; *p*—significance level; * small effect size; ** medium effect size; *** large effect size; ^a^ significant difference between groups (boys NS-boys S); ^b^ significant difference between groups (girls NS-girls S); ^c^ significant difference between groups (boys NS-girls NS); ^d^ significant difference between groups (boys S-girls S).

**Table 3:** The predictors of meeting the PA recommendations (11,000 steps/day and 60 min MVPA) among boys and girls who are non-sedentary and sedentary in classes.

Variables	Category	At Least 11,000 Steps/Day				At Least 60 Min MVPA (≥3 METs)			
		Boys		Girls		Boys		Girls	
		*OR* (95% CI)	*p*	*OR* (95% CI)	*p*	*OR* (95% CI)	*p*	*OR* (95% CI)	*p*
Model 1									
Sedentary	Most	Ref.							
	More	1.33 (0.73–2.43)	0.351	1.49 (0.99–2.26)	0.059	2.01 (1.12–3.62)	0.020	1.47 (0.97–2.23)	0.072
	Less	1.54 (0.85–2.81)	0.158	1.40 (0.92–2.13)	0.112	1.75 (0.97–3.18)	0.065	1.35 (0.89–2.06)	0.165
	No	2.26 (1.26–4.06)	˂ 0.001	2.47 (1.65–3.71)	˂ 0.001	3.46 (1.92–6.21)	˂ 0.001	1.80 (1.19–2.72)	0.005
Model 2									
Sedentary	Most	Ref.							
	More	1.35 (0.73–2.50)	0.335	1.46 (0.96–2.22)	0.078	2.00 (1.10–3.65)	0.023	1.41 (0.92–2.15)	0.115
	Less	1.59 (0.86–2.91)	0.137	1.39 (0.92–2.12)	0.123	1.77 0.97–3.25)	0.063	1.35 (0.88–2.06)	0.172
	No	2.38 (1.30–4.34)	0.005	2.47 (1.64–3.71)	˂ 0.001	3.62 (1.98–6.63)	˂ 0.001	1.82 (1.20–2.76)	0.005
Age	<16	Ref.							
	16	1.47 (0.88–2.45)	0.144	0.98 (0.70–1.38)	0.916	1.81 (1.08–3.03)	0.024	1.21 (0.85–1.72)	0.285
	≥17	1.01 (0.57–1.79)	0.981	0.95 (0.65–1.37)	0.776	1.54 (0.88–2.72)	0.131	1.42 (0.97–2.07	0.071
BMI	<18	Ref.							
	18–24.9	1.24 (0.51–3.02)	0.632	1.58 (0.93–2.68)	0.094	0.73 (0.31–1.69)	0.461	1.84 (1.04–3.22)	0.035
	≥25	1.27 (0.48–3.34)	0.635	1.52 (0.81–2.87)	0.197	0.65 (026–1.65)	0.364	1.45 (0.74–2.85)	0.275
Country	Czech Republic	Ref.							
	Poland	0.94 (0.42–2.11)	0.884	0.64 (0.35–1.17)	0.144	0.69 (0.31–1.56)	0.373	0.55 (0.29–1.02)	0.060

MPVA = moderate-to-vigorous physical activity; BMI = Body Mass Index; *OR* = odds ratio; *CI* = confidence interval; *p* = level of statistical significance; Model 1 = Sedentary; Model 2 = Adjusted for age, BMI and country.
